# Fiber Optic Gas Sensors Based on Lossy Mode Resonances and Sensing Materials Used Therefor: A Comprehensive Review

**DOI:** 10.3390/s21030731

**Published:** 2021-01-22

**Authors:** Ignacio Vitoria, Carlos Ruiz Zamarreño, Aritz Ozcariz, Ignacio R. Matias

**Affiliations:** 1 Electrical, Electronic and Communications Engineering Department, Public University of Navarra, 31006 Pamplona, Spain; ignacio.vitoria@unavarra.es (I.V.); aritz.ozcariz@unavarra.es (A.O.); natxo@unavarra.es (I.R.M.); 2 Institute of Smart Cities (ISC), Public University of Navarra, 31006 Pamplona, Spain

**Keywords:** gas sensor, lossy mode resonance, optical fiber, ammonia, volatile compounds, humidity

## Abstract

Pollution in cities induces harmful effects on human health, which continuously increases the global demand of gas sensors for air quality control and monitoring. In the same manner, the industrial sector requests new gas sensors for their productive processes. Moreover, the association between exhaled gases and a wide range of diseases or health conditions opens the door for new diagnostic applications. The large number of applications for gas sensors has permitted the development of multiple sensing technologies. Among them, optical fiber gas sensors enable their utilization in remote locations, confined spaces or hostile environments as well as corrosive or explosive atmospheres. Particularly, Lossy Mode Resonance (LMR)-based optical fiber sensors employ the traditional metal oxides used for gas sensing purposes for the generation of the resonances. Some research has been conducted on the development of LMR-based optical fiber gas sensors; however, they have not been fully exploited yet and offer optimal possibilities for improvement. This review gives the reader a complete overview of the works focused on the utilization of LMR-based optical fiber sensors for gas sensing applications, summarizing the materials used for the development of these sensors as well as the fabrication procedures and the performance of these devices.

## 1. Introduction

During the past few decades, a lot of effort has been dedicated to detecting the concentration of gases due to the wide and relevant applications where this action is needed [1]. Gas sensors are necessary for the monitorization of the air quality in the environment, controlling production processes in industry, diagnosing diseases, or alerting the public to dangerous and toxic gases, among others. In recent years, there has been an increase in the demand for better sensors due to the appearance of new and more restrictive environmental laws that control the air pollution in our cities [2]. The detection limits of these laws are lowering the gas concentrations, creating a need for more sensitive sensors. Additionally, trends like the Internet of Things or industry 4.0 create new demands in monitoring the parameters of the manufacturing processes, especially in chemical industries [3], giving also an opportunity to new gas sensing devices to be applied in these areas for the first time.

The applications for gas sensors are wide and heterogeneous. For example, gases in industrial processes are measured, such as NO_x_ in coal power plants, H_2_S in wastewater treatment plants and ethanol in winemaking. Gas sensors also have relevance in the wellbeing of humans, as they monitor dangerous gases including volatile compounds (VCs) that are the main pollutants of indoor airs and NO_x_ gases that cause respiratory and neurological disorders. Furthermore, gas sensors are employed in the healthcare industry; various gases are biomarkers of diseases, like ammonia, which is used for detecting liver dysfunction, or acetone, which is used for detecting diabetes.

In general, gas sensing methods rely on the modification of the physical/chemical properties of the gases in their interactions with other materials like semiconductor metal oxides, polymers and carbon nanotubes [1]. In this sense, there is a wide variety of gas sensors, which can be classified according to the detection method used, such as calorimetry [4], gas chromatography [5,6], acoustic [7], optical [8] as well as other methods based on the measurement of the electrical properties of the materials [1].

A particular case consists of optical fiber gas sensors, which are small and flexible, appropriated for confined spaces and immune to electromagnetic interferences. They are also resilient to difficult atmospheres with high temperatures, high humidity or dangerous concentrations of corrosive or explosive gases. These advantages enable their utilization in niche applications where no other technology can be used [8,9]. In addition, the use of optical fiber allows not only the transmission of light to remote zones, but also the use of optical fiber itself as the sensing part with easy multiplexing capabilities or even the possibility to perform distributed measurements. Particularly, optical fiber gas sensors have been obtained using different optical fiber configurations, such as single mode fiber (SMF) [10], multimode fiber (MMF) [11,12], D-shape fiber [13] or photonic crystal [14] as well as diverse interrogation techniques, such as nondispersive infrared sensors (NDIR) [15], interferometry [16], long periods fiber gratings LPFG [17] or resonances [18], among others [8].

Optical fiber resonance-based sensors are generally obtained using thin nanofilms or nanostructures fabricated onto the cylindrical waveguide. Here, the well-known Surface Plasmon Resonance (SPR) sensors have been stablished as a gold standard since the development of the first optical fiber sensor in 1993 [19]. These devices enable the gas molecules to interact with the thin-film, originating a shift in the resonance wavelength, which can be directly correlated with the gas concentration [18]. A different type of resonance-based optical fiber sensors consists of lossy mode resonance (LMR) based sensors. LMR-based optical fiber sensors have gained popularity during the last decade owing to their similarities with SPR. However, LMR-based optical fiber sensors stand out from other resonance techniques due to their advantages like the use of a wide variety of sensing materials or the lack of a need for polarized light. One of the main differences between SPR and LMR is associated to the material that produces the phenomenon, which is discussed in detail in Section 2. Nevertheless, it is important to note that SPR sensors are limited to the use of metals like gold, silver or platinum, whereas LMR are obtained with metal oxides or polymers, providing a greater versatility. Additionally, oxide metals like TiO_2_ [20], WO_3_ [21], SnO_2_ [22], ZnO [23] or graphene oxide [24], among others, have proven to be sensitive to different gaseous species, which enhances the possibilities of the utilization of LMR-based optical fiber gas sensors over SPR-based optical fiber gas sensors.

The aim of this review consists of drawing attention to the possibilities of the utilization of optical fiber LMR-based devices for gas sensing as well as providing a comprehensive review of the promising results obtained in this field using this interrogation technique. This paper includes works where the LMR phenomena is exploited for gas sensing applications, as well as other papers where some interesting gas sensing materials with high potential to be used in the development of LMR-based optical fiber gas sensors are described.

The review is structured as follows: The introduction details the importance and variety of gas sensors and gas sensing technologies, respectively, with special attention to optical fiber gas sensors based on LMR. The next section describes in detail the LMR phenomenon as well as its differences and similarities with SPR. The subsequent sections describe the utilization of LMR-based optical fiber gas sensors for the detection of particular gaseous species, starting with ammonia, followed by water vapor (better known as humidity sensors), a compilation of volatile compounds (VCs), such as ethanol, methanol and acetone, and finishing with an examination of other gases, including hydrogen sulfide, hydrogen and nitrogen oxides. The review concludes with an overview of the works in this field as well as an outlook of the possibilities of LMR-based optical fiber sensors for gas sensing applications.

## 2. Fundamentals of Lossy Mode Resonances (LMR)

A decade ago, the first LMR sensor was published [25]. Since this work, multiple papers have showed the potential of this type of sensor [26], measuring refractive index of the medium [27], humidity [28], pH [29], chemical compounds [30], voltage [31] or biological material (DNA chains, proteins, biomarkers, etc.) [32]. The LMR phenomenon is not as studied as the SPR phenomenon but it has gained attention during the last decade and the number of papers in this novel field is increasing year after year, reaching nearly 500 entries in the Scopus database, which is indicative of its potential.

Resonance-based sensors are generally obtained using thin-films or nanostructures fabricated onto the optical fiber. In particular, the LMR phenomenon is originated when a guided mode in the optical fiber experiences a transition to guidance in the thin-film. The transition generates a maximum attenuation band at a specific wavelength, known as resonance wavelength. When a second mode is guided in the thin-film, another resonance wavelength is obtained and this can continue with other modes, causing higher order resonances to appear [26]. The transitions of the modes are less abrupt with higher orders, so the resonances are less sensitive than the previous ones. In addition, the higher the order of the LMR, the shorter the resonance wavelength. The center wavelength of the resonances is highly sensitive to the characteristics of the structure (e.g., the thickness of the thin-film, the refractive index of the optical fiber, thin-film and surrounding medium) [33]. Therefore, physical changes in the sensor can induce resonance wavelength shifts in the spectrum. Due to this condition, the LMR phenomenon is suitable to be used for sensing applications measuring different parameters depending on the characteristics of the structure.

The optimization of the LMR for sensing applications can be achieved attending to three main parameters: the refractive index of the film, the film thickness and the refractive index of the surrounding medium. There can be sensors that detect the changes of the refractive index of the film, the modifications of the film’s thickness or the refractive index of the medium. In addition, a parameter can be changed as desired in order to control the wavelength of the resonance wavelength; for example, the thickness can be easily changed in the fabrication process to tune the LMR in a desired zone of the spectrum.

The LMR phenomenon has some advantages over other optical fiber sensing technologies such as LPFGs. There is no need for the use of gratings, so the fiber is simpler. Moreover, the wavelength shift is more abrupt and linear than the LPFGs sensors [34].

**Figure 1:**
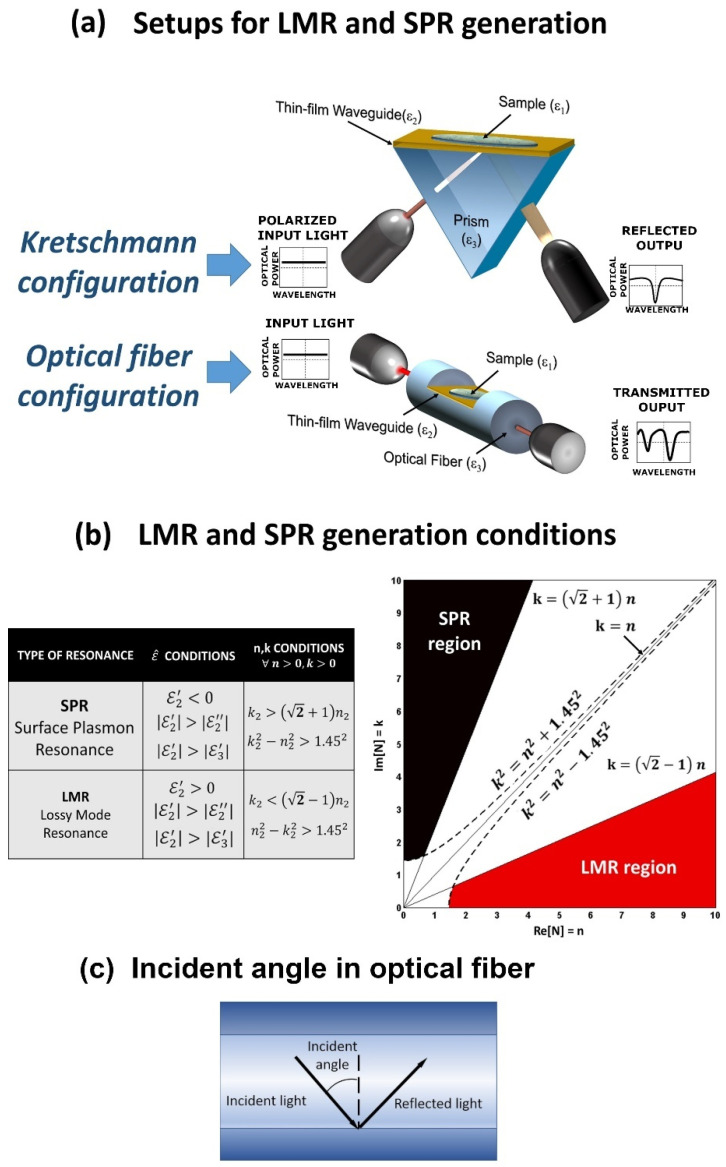
(**a**) Structure of Kretschmann configuration and D-shaped optical fiber; (**b**) conditions of the material for the generation of lossy mode resonance LMR and surface plasmon resonance SPR; (**c**) schematic of the incident angle in an optical fiber [26]. Reprinted from Sensors and Actuators B: Chemical, 240, Del Villar et al., Optical sensors based on lossy-mode resonances, 174-185, Copyright 2016, with permission from Elsevier.

SPR and LMR have a lot of similarities. They share similar structures in which it can be observed that their resonance has similar shapes in the spectrum, and they are sensitive to the refractive index of the medium. In fact, SPR and LMR can be easily mistaken. Both SPR and LMR can be obtained using similar configurations, as it is represented in Figure 1a. The Kretschmann configuration consists of a more mature setup that utilizes a nanocoated optical prism, whereas the optical fiber configuration uses an optical fiber. Regarding the optical fiber configuration, typical setups are based on a MMF where part of the cladding has been removed (CRMMF), a tapered fiber or a D-shape fiber (see the bottom of Figure 1a). Recently, the LMR phenomenon has been also demonstrated using nanocoated planar waveguides, such as slides or coverslips [35].

LMR-based sensors can be implemented measuring the shift of the resonance wavelength or monitoring the intensity associated with the resonance wavelength peak shift. The wavelength shift method needs expensive equipment, such as spectrometers or similar and broadband light sources. In contrast, the intensity-based interrogation setups use more simple equipment (e.g., power meters or lasers) but they are sensitive to perturbations in the optical power intensity, such as movements in the fiber or instabilities of the light source.

As it was mentioned in the introduction, a major difference between SPR and LMR is associated with the materials used for the thin-film to generate the resonances. SPR is obtained when the real part of the permittivity is negative and higher in magnitude than its own imaginary part and that of the surrounding material. In contrast, the LMR is obtained when the real part of the permittivity is positive and higher in magnitude than its own imaginary part and that of the surrounding material, see Figure 1b. Materials that comply with the LMR conditions are metal oxides, polymers or combinations of the former, while the generation of SPR is usually limited to good conductors such as gold and silver. Here, it is noteworthy to mention that some materials may support both SPR and LMR phenomena. For example, indium tin oxide (ITO) satisfies the requirements for LMR and SPR generation in different regions of the spectrum simultaneously, as has been described in several works [25,36,37].

Another difference is the optimal angle of incident light (the angle between the incident light and the orthogonal of the surface where the light is reflected, see Figure 1c). A previous study shows that the angles between the sensing surface and the light in the range of 40–75° have the best performance for SPR [38], whereas LMR have their optimal angles near 90° [39]. Therefore, optical fiber configuration is more appropriated for LMR generation as well as being more compact and easier to integrate in gas chambers or difficult access zones than the bulkier Kretschmann configuration.

Another aspect where LMR differs from SPR is that SPR interrogation generally demands the utilization of polarized light, whereas for LMR it is not strictly necessary and makes it possible to disregard the utilization of polarization controllers and hence reduce the complexity and price of a final device. On the other hand, the utilization of polarized light with LMR sensors permit the obtaining of sharper resonance peaks and better performances by means of separating the two LMR induced by the transverse electric (TE) and transverse magnetic (TM) modes, as described in [40]. However, many other works using LMR have shown good performance with non-polarized light [41,42]. Another method for improving the full width at half maximum (FWHM) of LMR based devices is by nanocoating laser ablation, as demonstrated in a reflection configuration in [43].

Regarding the utilization of LMR-based optical fiber sensors for gas sensing applications, they can take advantage of the use of optical fiber as an optimal structure that allows the measuring of gases in remote places and harsh environments. Concerning temperature cross sensitivity, it can be considered negligible for small temperature fluctuations (a few degrees Celsius). However, a proper temperature control is required in order to improve the signal to noise ratio or for applications in environments with large temperature variations (tens of degrees Celsius) [44]. LMR sensors are robust against temperature variations compared to other sensing technologies, which could be associated to the nanometer thickness of the LMR-generating thin-films or the utilization of silica optical fibers. Furthermore, many of the materials (mainly metal oxides) that can be used for LMR generation (thin-films) are also sensitive to gases. Superior properties of LMR-based optical fiber sensors for gas sensing applications are demonstrated in the next sections of this review. The sensors described in this work are classified in different sections depending on the gas target for a better understanding and comparison of the devices, covering ammonia (NH_3_), water vapor (relative humidity) H_2_O, volatile compounds (VCs) and other gaseous species such as hydrogen sulfide H_2_S, hydrogen H_2_ and nitrogen oxides NO_x_.

## 3. Ammonia Sensors

Ammonia (NH_3_), is a colorless toxic gas with a characteristic pungent smell. It is used in the production of explosives, fertilizers and industrial coolants [45]. Studies have shown that ammonia gas contributes to the acidification and eutrophication effect in the environment [46]. In addition, it has an important impact on human health. The Occupational Safety and Health Administration (OSHA) recommends levels of ammonia lower than 25 ppm [47]. Concentrations above 35 ppm could lead to acute respiratory diseases in the human body [47]. Ammonia is released from slightly alkaline blood and expulsed through the skin or the breath. An increase in the ammonia concentration in breath or urine could be produced by a dysfunction in the kidney or liver, so it is a good indicator for early diagnostics of liver or stomach diseases [48]. For these reasons, there has been a huge effort to create high sensitivity ammonia sensors. In this section, a description of the most relevant studies found in this area are discussed. Although ammonia is a VC and there is a dedicated section for these gases, the large number of articles on this topic and the relevance of ammonia are sufficient to differentiate it in its own separate section.

There is a wide variety of techniques for ammonia detection [49]. The most widespread are the solid-state sensing methods which detect electrical changes in the sensing material, usually based on metal oxide and conducting polymers [49,50]. They share a simple and cost-effective fabrication method as well as a high sensitivity to NH_3_. However, their main disadvantages are their low selectivity and, for most of them, the operation at high temperatures, which increases the energy consumption. In particular, one of the highest sensitivity sensors of this type can detect concentrations of 100 ppb at room-temperature with response and recovery times of 7 s and 10 s, respectively [51].

Another well-known technique that makes it possible to obtain devices with high sensitivity and selectivity is tunable diode laser spectroscopy [49]. As an example, this technique demonstrated the measurement of ammonia concentrations at 150 pptv (part per trillion volume) operating at 10 Hz [52]. A drawback of this method is the fact that noise can easily deteriorate the performance of the sensors, so they need to be fabricated using highly accurate and stable components, which increases the cost. Other techniques used for ammonia detection are electrochemical sensors [53], surface acoustic wave [54] and field effect transistor sensors [55], among others [49].

Focusing again on optical fiber sensors based on LMR, which is the aim of this review, we can find several examples, which are summarized in Table 1. The works mentioned in Table 1 are either based on the LMR phenomenon or have potential to be used with this technology. These approaches are very heterogeneous, highlighting the ample possibilities of these devices, and demonstrating the utilization of a wide variety of sensing materials, optical structures and interrogation techniques, as described in the following paragraphs.

Ammonia dissolved in water detection was proven successfully by Divya Tiwari et al. using a LMR-based thin-film coated tapered optical fiber [46]. Here, TiO_2_ is used as coating material with porphyrin as functional material. The liquid phase deposition method (LPD) is used for the fabrication of the coating. Concentrations of ammonia gas between 0.1 and 10,000 ppm interacted with the deposited film, changing the refractive index of it and causing a displacement in the center wavelength of the LMR (resonance wavelength) of approx. 90 nm in the VIS-NIR region. Different configurations of tapered fiber were also fabricated in a boron-germanium co-doped optical fiber (SM750). The best results were obtained with a 17 µm diameter adiabatic tapered optical fiber achieving a lowest detection limit of 0.1 ppm with a time response of less than 30 s.

An optical transmission setup where the sensing material was deposited onto a CRMMF was exploited by Shajahan Shanavas et al. for ethanol and ammonia detection [56]. A spectrometer collects the output light, establishing a relationship between the spectrum of the light in the VIS and NIR region and the concentration of the target gases. Here, a nanocomposite formed by a CeO_2_ rice like nanostructure and multiwall carbon nanotubes prepared by the hydrothermal technique [57] is used as sensing material. This structure makes it possible to achieve a huge surface area of 108 m^2^ per gram of the nanocomposite with a 12.71 nm pore size. This permits the enhancement of the interaction with the gas molecules and enables a high sensitivity to be obtained (78 count/ppm for ethanol and 48 count/ppm for ammonia with concentrations between 0 and 500 ppm). In the case of ammonia gas, it has rapid response and recovery times of 28 s and 19 s, respectively, as well as a good stability with a variation of approx. 3.5% of the sensitivity in a 60 day test. However, the obtained devices do not have good selectivity and gases like acetone, toluene, methanol or ethane can induce similar responses as ammonia or ethanol. It is important to point out that the magnitude count is an arbitrary unit and depends on the instrument configuration. So, the comparation of the sensitivity with other works cannot be undertaken.

A miniature interferometry-based optical-fiber ammonia gas sensor was demonstrated by Yi Zhu et al. in [58] using a combination of optical fibers and tapered optical fibers (SMF-MMF-TAPER-MMF-TAPER-SMF). An optical spectrum analyzer and a broadband light source (1510–1560 nm) was used to measure the shift of the spectrum in relation to the concentration (0 to 5460 µg L^−1^) of ammonia in the gas chamber. The sensing material consisted of ZnO nanoflowers deposited on the sensing area (middle MMF and tapers) with a drop of a ZnO solution and 6 h drying in a vacuum oven at 60 °C. Ammonia sensitivity performance is compared between ZnO nanoflowers and ZnO nanospheres of approximately the same size (1 µm) showing sensitivities of 5.75 pm/(µg·L^−1^) versus ~2.25 pm/(µg·L^−1^), respectively. In addition, ZnO nanoflowers shown a better selectivity for gases like ethanol acetone methanol o methylbenzene. The cross sensitivity with the humidity is also studied, concluding that the sensors response can be almost independent of the humidity in the range of 35 to 60%RH.

**Figure 2:**
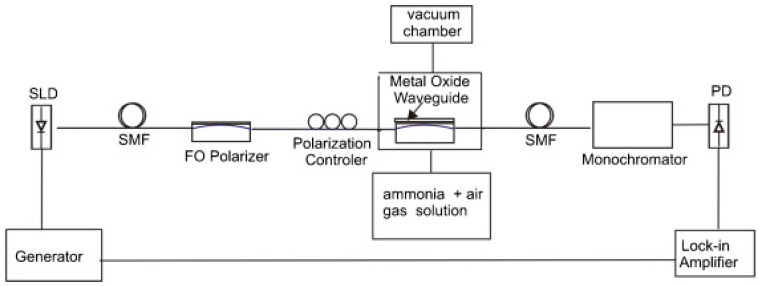
Optical setup of an ammonia sensor using a D-shape single mode fiber SMF and ZnO film as sensing material [59]. Reprinted from Sensors and Actuators B: Chemical, 146, Dikovska, A.O. et al., Optical sensing of ammonia using ZnO nanostructure grown on a side-polished optical-fiber, 331-336, Copyright 2010, with permission from Elsevier.

A similar work using ZnO as sensing material is also presented by A.OG. Dikovska et al. in [59]. In this case, three different films of ZnO were studied. The films were classified as smooth, porous and nanostructured. The sensor was formed by a side-polished single-mode fiber, known as D-shaped fiber (D-shaped fiber is obtained polishing a SMF and removing a side of the cladding until the core is almost exposed), with a nanostructured ZnO thin-film fabricated onto the plane region. The fiber was glued to a fused quartz block, and a ZnO layer, lower than 30 nm in thickness, was grown on the top of the sensor by the pulsed laser deposition (PLD) technique [60] in two steps (first, on-axis PLD configuration, and second, off-axis PLD configuration at a distance of 1.5 cm). The interrogation setup (see Figure 2) consists of a transmission setup with polarized light from a SLD light source where a photodiode in combination with a monochromator collect the output light. The resonance shift is monitored with concentrations of ammonia between 500 and 5000 ppm with the three different films. The best results were obtained with the nanostructured film that shown a 6.35 nm resonance shift between 0 and 5000 ppm (2.14 nm and 0.81 nm shift was obtained for porous and smooth films, respectively) and a detection limit of 50 ppm.

A different approach is presented by T. Kavinkumar et al. using graphene oxide (GO) as sensing material [61]. Here, three types of GO obtained using different annealing procedures (no treatment, heat treated at 110 °C in a vacuum atmosphere and treated at 220 °C in a vacuum atmosphere) are compared. A detailed characterization of the material was elaborated using powder X-ray diffraction, Fourier-transform infrared, micro Raman and ultraviolet-visible-near infrared spectroscopy techniques, showing that thermal treatment changes the properties of the material and reduces the interlayer spacing of the GO, which is associated with modifications or disappearing of various oxygen functional groups, such as hydroxyl, carboxyl or epoxide. GO thin-films were obtained using the dip-coating technique with a plastic (PMMA) CRMMF of 750 µm diameter. Then, the fiber was introduced in a gas chamber and subjected to different concentrations of ammonia, ethanol and methanol (0–500 ppm). The change of the intensity in the spectrum was recorded in a transmission setup with a tungsten light source and a spectrometer (100–2000 nm). Untreated GO revealed the best sensitivity (−0.32 counts per ppm versus −0.26 of GO_110 °C_ and −0.20 GO_220 °C_). Furthermore, the hydrophobic properties of untreated GO could be advantageous for the detection of ammonia in aqueous media. Concerning the selectivity, the three sensors revealed similar responses to ammonia, ethanol and methanol gases but a negligible response against water vapor.

**Figure 3:**
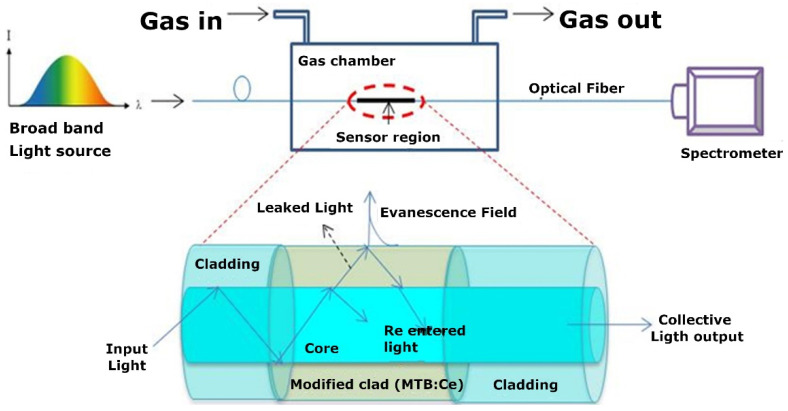
Schematic diagram of the sensor and the mechanism of gas sensing [47]. Reprinted from Sensors and Actuators A Phys. 285, Mohandoss, R. et al., Fiber optics assisted ammonia gas detection property of gamma irradiated magnesium tetraborate, 158-164, Copyright 2018, with permission from Elsevier.

A previous structure (PMMA CRMMF with an overall diameter of 750 µm and a numerical aperture NA = 0.5) was also employed by R. Mohandoss et al. using magnesium tetraborate (MTB) as the sensing material [47]. The sensing structure was fabricated using the slurry deposition method. This deposition technique consisted of mixing the sensing material with isopropyl alcohol that later dries, leaving the sensing material in the desired place. Different modifications of the sensing material have been explored such as cerium doped, glassy form and gamma irradiated. The setup of the sensor can be seen in Figure 3. Here, a halogen light source couples the light to the modified optical fiber and a spectrometer analyses the output visible light (200–1100 nm) as a function of the ammonia concentration (0–500 ppm). The spectral response is modified in amplitude with maximum intensity at 685 nm. The results show an increase in the sensitivity to ammonia gas in Cerium doped (0.25 mol.%) samples. Additionally, the samples irradiated with a dose of 20 Gy in a ^60^Co gamma chamber improve previous results, making the gamma irradiated MTB:Ce glass an efficient fiber optic based ammonia sensor with a sensitivity of 74 counts/50 ppm. This structure has great potential to be used in LMR based gas sensors.

B. Renganathan, co-author of the previous paper, has published multiple articles regarding gas sensors following a similar procedure and using different materials. In particular, ammonia gas sensors have been studied using a setup similar to the one represented in Figure 3 using nanocrystalline ZnO [62], nanocrystalline CeO_2_ [63], nanoparticles of V_2_O_5_ and nanoparticles of WO_3_ [64], nanocrystalline SnO_2_ and SnO_2_ film [65], nanocrystalline TiO_2_ [20], Zn_3_(VO_4_)_2_ nanopowder [66], nanocrystalline Sm_2_O_3_ [67] and single and multi-walled carbon nanotubes [68,69] fabricated by dip coating techniques [70]. Table 1 summarizes the described ammonia sensors and their most relevant parameters.

**Table 1:** Summary of performance and configuration parameters of NH_3_ sensors reviewed in Section 3.

Sensing Layer	Deposition Technique	Optical Waveguide	Gas Concentration	Sensitivity	Detection Limit	Response Time	Recovery Time	Reference
MPyP/TiO_2_	Liquid phase deposition method	taper of doped optical fibre (SM750)	0.1 to 10,000 ppm	Nonlinear ~1.2 nm ppm^−1^ in the range 0.1–10 ppm.	0.16 ppm	30 s	N/A	[46]
Magnesium tetraborate, MgB_4_O_7_ (MTB)	Slurry deposition	PMMA 750 µm	0 to 500 ppm	1.48 counts ppm^−1^	50 ppm *	N/A	N/A	[47]
CeO_2_/Multi walled carbon nanotubes	Hydrothermal technique	PMMA 550 µm	0 to 500 ppm	0.096 counts ppm^−1^	50 ppm *	28 s	19 s	[56]
Three-dimensional ZnO nanoflowers	Drop coating	SM-MM-SM	0 to 7281 ppm	4.32 pm ppm^−1^	1210 ppm *	~26 s	~39 s	[58]
ZnO nanostructures	Pulsed laser deposition method	D-shape fiber	0 to 5000 ppm	~0.66 pm ppm^−1^	~50 ppm	N/A	N/A	[59]
Graphene oxide	Dip coating	PMMA 750 µm	0 to 500 ppm	−0.32 counts ppm^−1^	100 ppm *	N/A	N/A	[61]
Nanocrystalline ZnO	Dip coating	PMMA 750 µm	0 to 14 KPa	− 0.017 kPa^−1^	0.2 kPa *	100 min	80 min	[62]
Nanocrystalline CeO_2_	Dip coating	PMMA 750 µm	0 to 14 KPa	12 10^−3^ kPa^−1^	0.2 kPa *	64 min	51 min	[63]
Nanoparticles of V_2_O_5_ and WO_3_	Dip coating	PMMA 750 µm	0 to 14 KPa	4 10^−3^ kPa^−1^	0.2 kPa *	40 min	39 min	[64]
Nanocrystalline SnO_2_ and fim SnO_2_	Thermal evaporation	PMMA 700 µm	100 to 1000 ppm	0.09 counts ppm^−1^	100 ppm *	20 min	120 min	[65]
Nanocrystalline TiO_2_	Dip coating	PMMA 750 µm	50 to 500 ppm	0.6 counts ppm^−1^	50 ppm *	83 min	77 min	[20]
Zn_3_(VO_4_)_2_ nanopowder	Dip coating	PMMA 750 µm	20 to 500 ppm	0.019 µV ppm^−1^	50 ppm *	46.8 min	59 min	[66]
Sm_2_O_3_	Dip coating	PMMA 750 µm	0 to 500 ppm	14 10^−3^ kPa^−1^	1 kPa *	67 min	57 min	[67]
Single and multi-walled carbon nanotubes	Dip coating	PMMA 750 µm	0 to 500 ppm	0.31 counts ppm^−1^	50 ppm *	N/A	N/A	[68]
Single-walled carbon nanotubes	Dip coating	PMMA 750 µm	0 to 500 ppm	1.3 counts ppm^−1^	12.5 ppm *	60 min	50 min	[69]

* Data not specified. The values are the minimum concentration found in the articles.

## 4. Humidity Sensors

Sensors for water vapor gas concentration, popularly known as humidity sensors, are essential in many fields, such as industry, agricultural processes, air quality monitoring, air conditioning, manufacturing processes, control and storage among others [71]. In order to fulfil these needs, sensors with high sensitivity, wide humidity detection range, rapid response and short recovery times are required [24]. In this sense, humidity sensors have been thoroughly studied using LMR-based optical fiber sensors. This section reviews the fabrication and performance of different LMR-based humidity sensors, which are also summarized in Table 2.

Humidity is the magnitude that specifies the amount of water vapor in a mix of gases (usually air). The most common expression for this magnitude is relative humidity, although it can also be expressed as absolute humidity. The relative humidity is a percentage that depends on the temperature. It is the ratio of the partial pressure of water vapor in a mix of gases to the saturation vapor pressure in that mix at the same temperature. In other words, is the amount of water vapor as a percentage of the total amount that could be held at that temperature. In contrast, absolute humidity is independent of the temperature and it is expressed with the ratio of mass or volume between the water vapor and the other gases. For example, it is used parts per million (ppm) by volume fraction or ratio of the molecular weight or grams per cubic meter (g/m^3^).

Due to the high importance of this type of measurement and its large number of applications, different technologies have been developed. Leaving aside the measuring technique, relative humidity (RH) sensors can be classified attending to the materials used for humidity sensing [71]. Ceramic materials (Al_2_O_3_, TiO_2_, SiO_2_ or spinel compounds) change their conductivity and dielectric constants with changes in humidity. Semiconductor materials, such as SnO_2_, perovskite compounds or In_2_O_3_, increase or decrease the conductivity of the *n*-type and *p*-type semiconductors, respectively, with the rise in humidity. Additionally, polymer-based sensors change their resistance, conductivity or capacitance with changes in humidity. In the case of absolute humidity, devices like hygrometers or aluminum oxide moisture sensors are used for the measurements.

The utilization of optical fiber sensors for humidity measurements has been widely reviewed by different authors using diverse optical configurations and sensing materials, such as red doped polymethylmethacrylate [72], CoCl_2_ doped thin polymer [73], Rhodamine B/hydroxypropyl cellulose [74], thin polyvinyl alcohol/CoCl_2_ [75] or porous sol–gel silica (PSGS) [76].

However, the first optical fiber humidity sensor based on LMRs was reported by C. R. Zamarreño et al. in [77]. The sensing material was deposited onto a CRMMF and was formed by an ITO thin-film and a nanostructurated multilayer polymeric film. The dip-coating fabrication technique was used for the ITO film in order to produce an optical fiber refractometer in the NIR region. Then, a multilayer composed of poly/allylamine hydrochloride (PAH) and poly(acrylic acid) PAA was built with the layer-by-layer technique. The thickness of the polymeric layer was sensitive to humidity, so changes in humidity produced a variation in the thickness and the refractive index of the surrounding medium, shifting the resonance wavelength. The equipment used to monitor the LMR was a DH-2000-H halogen light source and a NIR 512 spectrometer in a transmission setup. The manuscript compared two sensors with different numbers of bilayers of PAA-PAH. The first one with 100 bilayers was tuned to where the LMR was located in the most sensitive region of the spectrum (1400–1500 nm), and the other one with 20 bilayers was positioned in a less sensitive region (1000–1100 nm). An increase in humidity from 20% to 90%RH shifted the LMR peak 58 nm and 13 nm, respectively, with a sensitivity of 0.83 and 0.18 nm/%RH, respectively.

The same author implemented a humidity sensor using titanium dioxide (TiO_2_) and poly(sodium 4-styrenesulfonate) (PSS) as sensing material [78]. A CRMMF (FT200EMT, Thorlabs Inc., Bergkirchen, Germany), 200/225 μm core/cladding diameter optical fiber was coated with TiO_2_/PSS coating by the layer-by-layer technique. A transmission setup was implemented, with a halogen light source and two spectrometers covering the VIS and NIR region. The sensitive region of the sensor head was introduced in a climatic chamber where the humidity was changing between 20 and 90% RH. The resonance wavelength shift was due to the change in the refractive index of the coating caused by the penetration of water inside the film’s pores. The sensitivity of this device was 1.43 nm/%RH for the first resonance and 0.97 for the second one. The sensitivity could be differentiated in two regions, 20–70% and 70–90%, where different shift variations occurred, probably due to nonlinearities of the coating.

The previous research group also described the fabrication of optical fiber humidity sensors based on LMR using different materials such as ITO and agarose [79], SnO2 [80] and PAH-PAA [81].

The utilization of graphene oxide (GO) for the fabrication of optical fiber humidity sensors has been studied by Miguel Hernaez et al. in [24]. In this study, a multimode CRMMF of 200 µm of diameter (FT200EMT) was employed. The sensing structure comprises a first layer of SnO_2_ fabricated using the sputtering deposition technique and followed by a multilayer structure including Polyethylenimine (PEI) and GO fabricated using the layer-by-layer electrostatic assembly method. The first SnO_2_ layer generates the LMR at 420 nm while five additional bilayers of PEI/GO shift the resonance wavelength to 536 nm. The sensitivity of the device is 0.317 nm/%RH in the range 20–70%RH and 1.352 nm/%RH in the range 70–90%RH. The response time is 160 ms and the recovery time 262 ms. (time between 10% and 90% of the maximum wavelength shift and vice versa). In a more recent article [82], the same author has managed to build a LMR sensor without the need of the thin-film of SnO_2_, opening the path for a new humidity sensor based on only a GO film.

**Figure 4:**
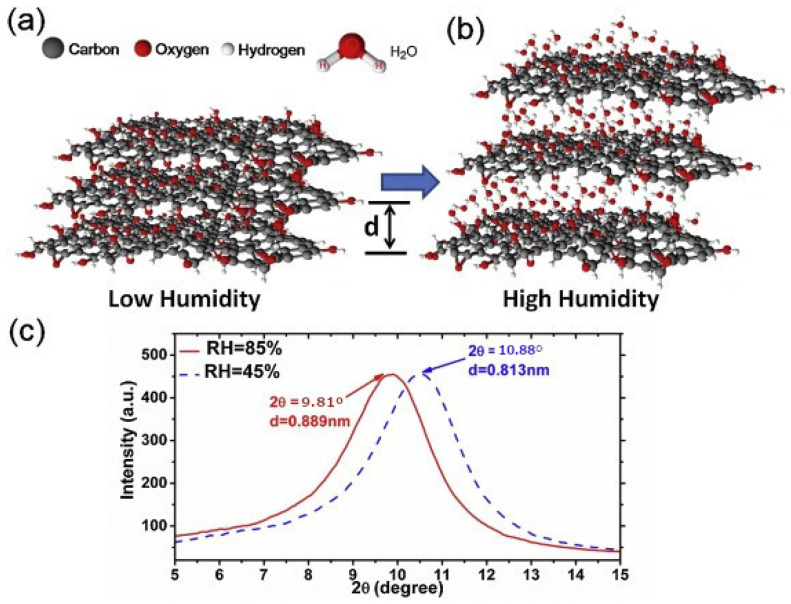
(**a**) Graphene oxide GO film with low humidity. (**b**) GO film with water molecules in high humidity. (**c**) X-ray diffraction (XRD) spectra with relative humidity of 45% and 85% [44]. Reprinted from Sensors and Actuators, B Chem. 255, Huang, Y. et al., High-performance fibre-optic humidity sensor based on a side-polished fibre wavelength selectively coupled with graphene oxide film, 57-69, Copyright 2017, with permission from Elsevier.

Another humidity sensor based on GO fabricated by spontaneous evaporation is explored by Yaoming Huang et al. in [44] using a D-shape optical fiber also known as side-polished SMF (SPF). Although the author does not describe the sensor as being LMR based, the type of material, setup and results are conclusive, so we can infer that it is most likely of this type. Therefore, it will be described as an LMR sensor in this review. The sensing principle relies on the change of the film thickness produced by the humidity. When the humidity increases, the nanopores of the film open and water molecules are introduced inserting themselves between the layers of the structure. In that situation, the H-bonding between the functional groups and the water molecules interlinking the adjacent layers is replaced by the H-bonding of water molecules. This change produces a swelling of the film, as it can be seen in Figure 4.

The sensor is placed in a transmission setup that consists of a tunable laser (from 1520 nm to 1620 nm) followed by a polarization controller previous to the sensitive region of the fiber and finishing with an optical spectrum analyzer (OSA) and an optical power meter. The OSA enables the measuring of the resonance wavelength shift, which is associated to the relative humidity variations. In the range of 32.04% to 85.00% RH, the sensitivity of the device was 0.138 nm/%RH and 0.145 nm/%RH for the first and second LMR, respectively. In the range of 85.0–97.6%, the sensitivity of the sensor was 0.897 nm/%RH and 0.915 nm/%RH for the first and second LMR, respectively. This abrupt change of sensitivity is attributed to the swelling effect where the H-boding of water contributed more significantly that the water-carboxylic group H-boding. The effect of the temperature is also studied, showing a change of 0.0027 nm/°C. The sensor has fast response and recovery times 2.73 and 7.27 s, respectively, which permits its utilization for human breath monitoring. A cost-effective approach to obtain the humidity value consists of substituting the OSA with an optical power meter. In this case, the change of the intensity of the light is measured using a 1585 nm laser, obtaining a linear correlation with RH and a sensitivity of 0.427 dB/%RH.

The utilization of D-shaped optical fiber for humidity detection is also described using tungsten disulfide WS_2_ as sensing material and light intensity measurements (using optical power meters and a 1550 nm laser) [83]. Here, a sensitivity of 0.1213 dB/% RH was obtained in a range of 35–85%. The sensor shows a fast response time (1 s) and recovery time (about 5 s), which reveals a high potential for human breath monitoring. Although this sensor is not based on the LMR phenomenon, the sensing material has great potential to be used in a LMR sensor, possibly allowing a new study in a humidity sensor based on the shifting of the resonance wavelength. Other works that describe the performance of LMR-based RH sensors are also summarized in Table 2 [84,85,86,87].

It is worth noting that the same sensitivity pattern has been observed in several works [24,44,78,87] where we can find two distinctive zones of RH sensitivity. Different theories try to explain this fact by associating it with H-bonding in the case of GO [24,44], nonlinearties in the case of the TiO_2_/PSS [78] or the swelling behavior in the case of polyelectrolyte films [88].

Finally, in a recent work, coverslips were deposited with copper oxide, thereby obtaining a LMR based humidity sensor with a broadband range from 400 to 1700 nm, which made it possible to observe the better performance in the NIR region compared to the visible region. In fact, the sensitivity to humidity was improved by more than a factor of two. The sensitivity of this sensor was later optimized with an additional layer of agarose, permitting the observation of a 10-fold sensitivity increase compared to the same device without agarose [89].

**Table 2:** Summary of performance and configuration parameters of relative humidity RH sensors reviewed in Section 4.

Sensing Layer	Deposition Technique	Optical Waveguide	Sensitivity	Response Time	Recovery Time	Reference
Indium tin oxide (ITO) and Polyallylamine hydrochloride (PAH) and polyacrylic acid (PAA)	Dip coating and Layer-by-Layer	Optical fiber 225/200 μm	0.82 nm/%RH	approx. 1870 s	approx. 5250 s	[77]
		FT200EMT				
Indium tin oxide (ITO) and agarose	Dip coating and Boiling water method	Optical fiber 225/200 μm	0.75 nm/%RH	N/A	N/A	[79]
		FT200EMT				
Tin oxide (SnO_2_)	Dip coating	Optical fiber 225/200 μm	0.107 nm/%RH	N/A	N/A	[80]
		FT200EMT				
Polyallylamine hydrochloride (PAH) and polyacrylic acid (PAA)	Layer-by-Layer	Optical fiber 225/200 μm	0.37 nm/%RH	N/A	N/A	[81]
		FT200EMT				
TiO_2_ and poly(sodium 4-styrenesulfonate) (PSS)	Layer-by-Layer	Optical fiber 225/200 μm	1.43 nm/%RH for 1° resonance 0.97 nm/%RH for 2°	N/A	N/A	[78]
		FT200EMT				
Polyallylamine hydrochloride (PAH) and polyacrylic acid (PAA)	Layer-by-Layer	Optical fiber 225/200 μm	0.51 nm/%RH First LMR	N/A	N/A	[28]
		FT200EMT				
Polymeric and Ag particles	Layer-by-Layer	Optical fiber 225/200 μm	0.943 nm/%RH	0.476 s	0.447 s	[84]
		FT200EMT				
Indium tin oxide (ITO) and In_2_O_3_	Layer-by-Layer	Optical fiber 225/200 μm	0.283, 0.133, 0.042 nm/%RH ITO, In_2_O_3_ TE and TM mode	N/A	N/A	[85]
		FT200EMT				
Tin oxide (SnO_2_)	Sputtering	Taper SMF 9/125 um Telnet RI	1.9 nm/%RH	1.52 s	approx. 1.52 s	[86]
Indium tin oxide (ITO)	Sputtering	Planar waveguide coverslips	0.0657 nm/%RH from 30 to 65%RH	N/A	N/A	[87]
			0.212 nm/%RH from 65 to 90%RH			
SnO_2_ polyethylenimine (PEI) and graphene oxide (GO)	Layer-by-layer and sputtering	Optical fiber 225/200 μm	0.317 nm/%RH from 20 to 70%RH	0.16 s	0.262 s	[24]
		FT200EMT	0.212 nm/%RH from 65 to 90%RH			
Polymeric film and gold nanorods	Layer-by-Layer	Optical fiber 225/200 μm	~3 nm/%RH from 20 to 45%RH	N/A	N/A	[88]
		FT200EMT	11.2 nm/%RH from 45 to 90%RH			
Graphene oxide (GO)	Spontaneous evaporation	Type D single mode fiber	0.145 nm/%RH from 32 to 85%RH	2.73 s	7.27 s	[44]
			0.915 nm/%RH from 85 to 97.6%RH			
Copper oxide (CuO), tin oxide (SnO_2_) and agarose	DC sputtering and boiling water method	Coverslips	0.636 nm/%RH from 30 to 90%RH	N/A	N/A	[89]

## 5. Volatile Compounds (VCs)

VCs are organic chemicals that evaporate at ordinary room temperatures. They are mostly toxic to human beings; they create irritation to the eyes, nose and respiratory tract [9]. The World Health Organization (WHO) recognized VCs as the most important pollutants of indoor air, affecting human health with concentrations higher than 100 µg/m^3^ [90]. VCs are harmful to the environment due to their high vapor pressure that leads to an industrial pollution. VCs are used in many fields; for example, ethanol has applications in areas, such as the chemical, biomedical and food industries, smart cities, wine-quality monitoring and breath analysis [91]. VCs are also exploited as biomarkers. For example, the presence of acetone in human breath above 0.8 ppm is an accepted biomarker for type-I and type-II diabetes [92].

VCs concentration can be determined using different sensing technologies [90]. In general, electrochemical sensors enable measurements in a range between 0 and 50 ppm with an approximate resolution of 0.1 ppm and a response time of 30 s. The principle of operation of the sensors is based on a redox reaction where the gas molecules produce an electric current. A different technique, known as nondispersive infrared sensors (NDIR) [15], consist of the utilization of an infrared source and a detector with an optical filter. Here, the gas target absorbs light radiation at a particular wavelength proportionally to its concentration. These sensors are best suited for higher concentration measurements (in the range of 1000 ppm) as well as for infrared absorbing VCs, such as methane. Semiconductor metal oxide sensors (SMOS), previously mentioned in Section 3 and Section 4, can also be used to measure VCs, such as hydrocarbons, alcohols, ethers and aromatic compounds, among others [90]. Pellistors can measure flammable gases when there is an increase in temperature originated by the reaction between the gas and hot coil of platinum [90]. Another technology is photoionization, where the molecules of the gas are ionized and an electrometer measures the concentration of the gas [90]. A remarkable type of sensor is those popularly known as electronic noses. They consist of a matrix of individual sensors (semiconductor, electrochemical or PID) where the combined information can form a pattern for accurate measurements of a multiplicity of VCs. These sensors have been used for detecting diseases with the presence of VCs in breath [93] or urine [94], among others. In addition, other optical fiber sensors such as LPFGs have been implemented, measuring methane [95] and butane [96], to mention a few. The following paragraphs will describe some of the most remarkable works on optical fiber gas sensors based on LMR, which will be summarized with others in Table 3.

Ethanol was detected using SnO_2_ and CuO as sensing material by R.N. Mariammal et al. [91]. Two types of probes were prepared by a co-precipitation method (SnO_2_, and SnO_2_ and CuO with an average crystal size of 10 nm and 8–20 nm, respectively). The sensing material was embedded in a clad-modified fiber optic and the response was measured in the visible spectrum for ethanol concentrations between 0 and 500 ppm. SnO_2_ and SnO_2_:CuO exhibited a sensitivity of 13 and 41 counts/100 ppm, respectively. Higher sensitivity of SnO_2_:CuO is attributed to the high crystallinity, more oxygen vacancies and heterojunction formation of the structure. Although the sensor is not based on the LMR phenomenon, the sensing material is suitable for being used in LMR sensors.

An ethanol gas sensor was also studied by S. Vijayakumar et al. in [97] using two different probes (CRMMF with WO_3_ and a WO_3_/graphitic carbon nitride nanocomposite as sensing material). Fabricated devices were subjected to ethanol concentrations in the range 0–500 ppm and studied in a transmission layout in the region of 100–1100 nm. The nanocomposite revealed better sensitivity and selectivity than only WO_3_, which is associated with the increase in the composite crystallites’ size, surface area and pore volumes with the graphitic carbon nitride. Response and recovery times of 30 and 25 s, respectively, were obtained.

Cesar Elosua et al. [98] detected methanol, isopropanol and ethanol using an optical fiber LMR-based sensor with the organometallic [Au_2_Ag_2_(C_6_F_5_)_4_(NH_3_)_2_]_n_ compound as the sensing material and plastic clad silica PCS fiber as substrate. The layer-by-layer technique was used to fabricate the sensing film as well as control the thickness and, more importantly, the resonance wavelength position. The optical sensors were studied in the visible region in a transmission setup. Various LMRs were used in this work for VC detection, which increases the robustness of the sensor since the shift can be referenced to various resonance wavelengths at the same time. The sensitivity for methanol, isopropanol and ethanol was 0.131, 0.074 and 0.067 nm/ppm, respectively. The cross sensitivity of the device with temperature was negligible, while with relative humidity a 3.4% rate of error was found.

T. Wang et al. [99] obtained a methanol sensor by means of an organic–inorganic hybrid nanoporous film fabricated using the layer-by-layer technique. The film was composed by a multilayer structure of poly(allyamine hydrochloride) and silica nanoparticles (PAH/SiO_2_)n with an overlay of tetrakis(4-sulfophenyl)porphine (TPPS). A 200 µm core diameter fiber which was bent into a U-shape was studied in a transmission setup and in the visible region of the spectrum. The results revealed a high sensitivity with a reversible and reproducible response. It also shown a good selectivity to methanol with a change 4 and 16 times bigger than ethanol and 1-octanol, respectively, and a response time in the order of several minutes.

An isopropyl alcohol sensor was presented by Julie Charles et al. in [100]. Here, the sensing material was a polymer composite prepared trough polymerization of pyrrole and the formation of prussian blue nanocubes and TiO_2_ nanoparticles (PPy/PB/TiO_2_). The material was deposited onto a CRMMF by dip coating. The interrogation setup consists of a transmission setup in the visible region. The polymer composite sensor shows a higher sensing capacity (151 counts/100 ppm) than a sensor with only TiO_2_ (78 counts/100 ppm) for detecting isopropyl alcohol gas at room temperature in the range 0–500 ppm. Despite not being a LMR sensor, it could be the basis for one using the sensing material of this article.

VCs include a wide range of gases and the same sensing devices are usually tested with several VCs. Other sensors for VC detection were previously mentioned for ammonia detection in Section 3 and are also summarized in Table 3 [20,56,61,62,63,64,66,67,69]. Table 3 enumerates some of the main parameters of the reviewed devices for a better comparison by the reader. However, it must be taken into account that it is not possible to compare the sensitivity between devices from different studies when the light intensity (count/ppm) is measured. Since these measurements use an arbitrary unit (counts) and depend not only on the sensing structure and gas concentration but on the instrument configuration, a fair comparison is not feasible.

In this section, few sensors for VCs are based on the LMR effect. The novelty of the field opens the path to new discoveries with the use of the sensing materials described in this article. The author encourages other researchers to follow the path for new VCs sensors based on the LMR phenomenon since current works show good performance.

**Table 3:** Summary of performance and configuration parameters of volatile compounds sensors reviewed in Section 5.

Gas **	Sensing Layer	Deposition Technique	Gas Concentration	Sensitivity	Detection Limit	Optical Waveguide	Ref.
(2), (4), (1)	Nanocrystalline TiO_2_	Dip coating	50 to 500 ppm	0.6 (2), 0.35 (4), 0.29 (1) counts ppm^−1^	50 ppm *	Poly(methyl methacrylate) PMMA 750 µm	[20]
(1), (2)	CeO_2_/Multi walled carbon nanotubes	Hydrothermal technique	0 to 500 ppm	0.158 (1), 0.096 (2) counts ppm^−1^	50 ppm *	PMMA 550 µm	[56]
(2), (1), (4)	Graphene oxide	Dip coating	0 to 500 ppm	−0.32 (2), -0.26 (1), −0.2 (4) counts ppm^−1^	100 ppm *	PMMA 750 µm	[61]
(4), (1), (2)	Nanocrystalline ZnO	Dip coating	0 to 14 (2), 2.5 (1), 1.4 (4) kPa	210 (4), 190 (1), −170 (2) 10^−3^ kPa^−1^	~ 0.2 kPa *	PMMA 750 µm	[62]
(4), (1), (2)	Nanocrystalline CeO_2_	Dip coating	0 to 14 (2), 2.5 (1), 1.4 (4) kPa	71 (4), 12 (2), 5 (1) 10^−3^ kPa^−1^	~ 0.2 kPa *	PMMA 750 µm	[63]
(4), (1), (2)	Nanoparticles of V_2_O_5_ (a) and WO_3_ (b)	Dip coating	0 to 14 (2), 2.5 (1), 1.4 (4) kPa	(a): 56 (4), 4 (2) 10^−3^ kPa^−1^(b): 59 (4), 5 (2), 4 (1) 10^−3^ kPa^−1^	~ 0.2 kPa *	PMMA 750 µm	[64]
(2), (1), (4), (3)	Zn_3_(VO_4_)_2_ nanopowder	Dip coating	20 to 500 ppm	0.19 (2), 0.009 (1), 0.005 (4), 0.004 (3) µV ppm^−1^	50 ppm *	PMMA 750 µm	[66]
(2), (4), (1)	Sm_2_O_3_	Dip coating	0 to 14 (2), 2.5 (1), 1.4 (4) kPa	14 (2), 8 (4), 76 (1) 10^−3^ kPa^−1^	~1 kPa * (2)	PMMA 750 µm	[67]
					~0.75 kPa * (1)		
					~0.5 kPa * (4)		
(2), (1)	Single-walled carbon nanotubes	Dip coating	0 to 500 ppm	1.3 (2) 1.12 (1) counts ppm^−1^	12.5 ppm *	PMMA 750 µm	[69]
(1)	SnO_2_ (a) and SnO_2_: CuO (b)	Co-precipitation	0 to 500 ppm	−0.13 (a), −0.41 (b) counts ppm^−1^	50 ppm *	N/A	[91]
(3)	Magnesium tetraborate (MTB) (a), MTB doped with cerium (b), MTB doped with cerium after gamma irradiation (c)	Slurry deposition	50 to 500 ppm	0.04 (a), 0.54 (b), 0.7 (c) counts ppm ^−1^	50 ppm *	PMMA 750 µm	[92]
(1)	WO_3_/gC_3_N_4_ (a) and WO_3_ (b) nanocomposites	Dip coating	0 to 500 ppm	62.5 (a), 38.5 (b) % at 500 ppm	100 ppm *	PMMA 550 µm	[97]
(4), (9), (1)	Organometallic compound [Au_2_Ag_2_(C_6_F_5_)_4_(NH_3_)_2_] n	Layer-by-Layer	0 to 250 ppm	0.131 (4), 0.074 (9), 0.067 (1) nm ppm^−1^	25 ppm *	Plastic cad silica PCS fiber	[98]
(4)	(PAH/SiO_2_)n and (TPPS)	Layer-by-Layer	N/A	N/A	N/A	U-shaped PCS 200 µm	[99]
(6)	Polypyrrole/Prussian blue/Titanium dioxide composite (PPy/PB/TiO_2_) (a) and TiO_2_ (b)	Dip coating	0 to 500 ppm	1.51 (a), 0.78 (b) counts ppm ^−1^	50 ppm *	PMMA 750 µm	[100]
(1), (4), (5)	ZnO Nanorods	Dip coating	0 to 250 ppm	~6.5% (1), ~3% (4), ~1.2% (5) at 300 ppm	50 ppm *	PMMA 1000 µm	[101]
(1), (3)	Nanocrystalline MnCo_2_O_4_	Dip coating	0 to 500 ppm	0.11 (1), 0.068 (3) counts ppm^−1^	50 ppm *	PMMA 550 µm	[102]
(3), (2), (1)	Pristine (a) and amine functionalized (b) ZnO nanoflake	Dip coating	0 to 300 ppm	(a): 13 (3), 7 (2), 7 (5), 4 (1), 1.8 (7), 1.8 (4) %	30 (3), 20 (2), 10 (1), 10 (4), 10 (10), 10 (7) ppm	PMMA 1 mm	[103]
(4), (5), (7)				(b): 45 (2), 18 (7), 18 (5), 15 (3), 8 (1), 8 (4) %			
(3), (2), (1)	Nanocrystalline copper bromide (γ-CuBr)	Slurry deposition	0 to 750 ppm	5% (3), 0.75% (1), 0.42% (2) with 750 ppm	~50 ppm *	PMMA 750 µm	[104]
(3), (2), (1)	ZnO nanorhombuses	Dip coating	0 to 400 ppm	~24% (3), ~12% (2), ~4% (1) thiourea functionalized at 772 nm and 400 ppm	10 ppm *	PMMA 1000 µm	[105]
(3), (2), (1)	ZnO nanoparticles	No data	0 to 250 ppm	14% (3), 9% (2), 5% (1) with 250 ppm	50 ppm *	no data found	[106]
(3), (6), (8)	TiO_2_	Dip coating	0 to 500 ppm	−0.31 (3), −0.15 (6), −0.12 (8) counts ppm^−1^	50 ppm *	PMMA 750 µm	[107]
(1)	Nano-sized amorphous tin oxide SnO_2_	Electron-beam evaporation	1000 to 20,000 ppm	14% at 10,000 ppm	1000 ppm *	SMF	[108]
(1)	Undoped (a), Mn-doped (b) cobal ferrite CoFe_2_O_4_ nanoparticles	Chemical method	0 to 500 ppm	0.07 (a), 0.12 (b) counts ppm^−1^	100 ppm *	PMMA 750 µm	[109]
(4), (1), (2)	ZnO (a), Ce doped Zno (b), Al_2_O_3_ (c), CeO_2_ (d), Sm_2_O_3_ (e), WO_3_ (f), V_2_O_5_(g)	Chemical method	0 to 500 ppm	(2): −17 (a), 7 (b), 9 (c), 12 (d), 14 (e), −5 (f), 4 (g) 10^−3^ kPa^−1^	N/A	PMMA	[110]
				(4): 21 (a), −96 (b), 29 (c), 71 (d), 8 (e), 59 (f), 56 (g) 10^−3^ kPa^−1^			
				(1): −21 (a), −131 (b), 55 (c), −5 (d), 76 (e), −4 (f), 1 (g) 10^−3^ kPa^−1^			
(1)	Gd doped ZnO nanoparticles	Dip coating	0 to 1000 ppm	0.0552 counts ppm^−1^	100 ppm *	PMMA 750 µm	[111]
(1), (4), (9)	Organometallic compound [Au_2_Ag_2_(C_6_F_5_)_4_(NH_3_)_2_]n	Layer-by-Layer	0 to 140 (1), 150 (4), 200 (9) ppm	0.41 (1), 0.52 (4), 0.263(9) nm ppm^−1^	4 (1), 16 (4), 10 (9) ppm	PCS 200/220 um	[112]
(1), (4), (9)	Lithium tetraborate Li_2_B_4_O_7_	Gel method	50 to 500 ppm	−10 (1), −4 (4), 3 (2) counts ppm^−1^	50 ppm	PMMA 750 µm	[113]
(1)	Chunk-shaped ZnO nanoparticles	Thermal evaporation	0 to 500 ppm	3.5% for 500 ppm	100 ppm *	PMMA 700 µm	[114]
(3), (6), (8)	Nano-crystalline zinc oxide ZnO	Dip coating	0 to 500 ppm	0.009 (3), −0.001 (6), −0.0017 (8) counts ppm^−1^	50 ppm *	PMMA 750 µm	[115]

* Data not specified. The values are the minimum concentration found in the articles. ** (1) Ethanol, (2) ammonia, (3) acetone, (4) methanol, (5) hexane, (6) isopropyl alcohol, (7) chloroform, (8) benzene, (9) isopropanol.

## 6. Other Gases

### 6.1. Hydrogen Sulfide (H_2_S) Sensors

Hydrogen sulfide is a colorless, highly flammable and toxic gas with a smell of rotten egg. It is produced as a decomposition product or as a by-product in petroleum and oil refineries, wastewater treatment plants and paper mills. Humans can smell it at concentrations of less than 1 ppm and it is toxic at concentrations greater than 20 ppm. The gas causes irritation to the eyes and lungs, olfactory fatigue, and breathing difficulties [30]. The WHO reports that a few breaths at concentration of 700 mg/m^3^ can be fatal [116]. In addition, this gas plays an important role in mammals, being the third gasotransmitter discovered after NO and CO. H_2_S produces various signaling effects in almost every system such as the cardiovascular or central nervous system [117].

Due to the importance of this gas and its applications, a wide variety of technologies have been explored in order to determinate the concentration of the H_2_S. SMOS composed by materials like CuO, ZnO and SnO_2_ show good sensitivities [118]. Other technologies for detecting the gas are colorimetric dyes, ion-selective electrodes, chromatographic methods, fluorescence-based sensors and electrochemical detectors [117].

**Figure 5:**
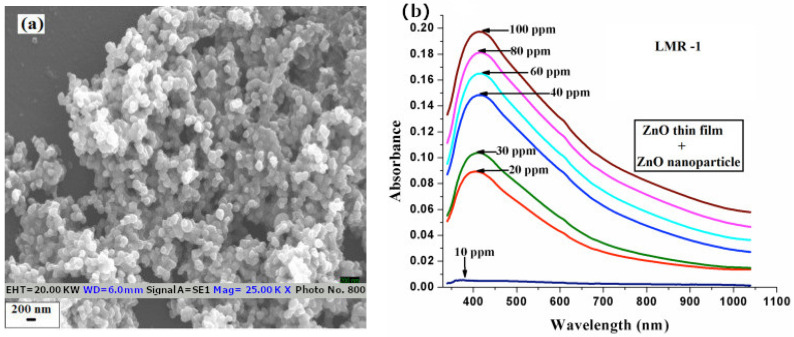
(**a**) Nanoparticles of ZnO; (**b**) shift in the resonance wavelength with different concentration of H_2_S [30]. Reprinted from Sensors and Actuators, B Chem. 255, Usha, S.P. et al., Fiber optic hydrogen sulfide gas sensors utilizing ZnO thin-film/ZnO.

A LMR-based optical fiber sensor for H_2_S has been developed by Sruthi P Usha et al. using various nanostructures of zinc oxide [30,119]. A plastic clad silica fiber with a core of 600 µm and a NA of 0.4 was used as substrate for the generation of the resonances in these devices. The sensors were arranged in a transmission setup and analyzed in the visible spectrum with a halogen light source and a spectrometer. The sensing part of the fibers were subjected to gas concentrations between 10 and 100 ppm (see Figure 5b). Resonance wavelength was monitored as a function of the gas concentration with all the fabricated devices. The results in [30] shown a sensitivity of 1.49 nm/ppm for the probe with a combination of ZnO thin-film (12 nm thickness) and ZnO nanoparticles (in the range of 40–50 nm), 1.06 nm/ppm for the probe with only ZnO nanoparticles and 0.24 nm/ppm with the sensing structure of 40 nm silver film and a 12 nm ZnO film (based on a SPR phenomenon in this case) (See Figure 5a). The results in [119] shown a sensitivity of 4.14 nm/ppm for the probe with only ZnO nanorods (150–200 nm radius and 26 of aspect ratio) and 2.35 nm/ppm for the probe of ZnO film (12 nm thickness) and ZnO nanorods.

### 6.2. Hydrogen H_2_ Sensors

Hydrogen is a colorless, odorless and inflammable gas (the range of combustion for hydrogen in air is 4.0–74.2 vol.%) [21]. Another important characteristic of this gas is its small molecule size, which facilitates the leaking of it. Due to these features, a rapid and accurate measurement of its concentration is needed in order to alert people to the formation of potentially explosive mixtures. It is present in various fields, such as hydrogenation processes, petroleum transformation, soldering or cryogenic cooling, among others. In addition, it is gaining popularity as a clean energy source. A wide variety of technologies can be used to measure this gas, such as catalytic, thermal conductivity, electrochemical or work function, among others [120].

An H_2_ optical fiber sensor based on LMR is proposed by Satyendra K Mishra et al., using indium tin oxide (ITO) [121]. A fiber of 600 µm core diameter and a NA of 0.37 was selected for the fabrication of the devices and arranged in a transmission setup that analyzes the visible region. Three probes with different coatings were subjected to concentrations of H_2_ between 0 ppm and 100 ppm. The sensitivities obtained from the probes were 0.71 nm/ppm for the combination of an ITO film (20 nm thickness) and spherical ITO nanoparticles (range of 60–80 nm), 0.58 nm/ppm for only ITO nanoparticles (60–80 nm) and 0.2 nm/ppm for only ITO film (20 nm thickness).

Another H_2_ sensor was developed with Zinc Oxide nanorods [122]. The gas was detected in a concentration of 0.25% at 150 °C. The fiber was a SMF where the cladding was reduced from 125 µm to 11 µm using hydrofluoric acid. The nanorods were synthetized by a growth solution technique. Response and recovery times are 7 and 3 min, respectively. The instruments used were an ASE light source and an optical spectrum analyzer in a transmission setup.

A hydrogen gas sensor based on a bent optical fiber coated with nanocrystalline SnO_2_ fabricated by sol–gel method was also presented in [123]. The sensing material, in contact with the hydrogen, caused a peak of around 320 nm in the absorption signal. Hence, the fiber was arranged in a transmission setup with a deuterium/tungsten light source and a UV/VIS spectrometer in order to detect the changes in the absorption. The sensor was able to detect high temperature hydrogen (300–800 °C) in a H_2_-N_2_ gas flow with concentrations from 0.1% to 10%. The response time was within seconds and the recovery time, when the gas flow was switched to compressed air, was 5 min.

WO_3_ was also used for hydrogen detection in [21]. Here, a CRMMF was coated with the WO_3_ film using a sol–gel method with a platinic acid as precursor and later calcined at 500 °C. The setup consists in a transmission configuration with a LED light source and a power meter. This work revealed a high sensitivity even at room temperature, with a 75% reduction in optical power of the fiber when it was exposed to 1 vol.% H_2_/99 vol.% N_2_. Although the sensor is not based on the LMR phenomenon, the sensing material has a great potential to be used in one, as it is explained in [124].

### 6.3. NO_X_ Sensors

Nitrogen oxide (NO_x_) is a family of gases usually obtained in combustions. They are a by-product of burning fossil fuels in vehicles and coal-powered thermal plants. The NO_x_ family produces environmental issues like summer smog or acid rain and it is also responsible for the destruction of the ozone layer. NO_x_ is harmful at 100 ppm concentration, causing respiratory and neurological disorders [125]. Due to these negative effects, efforts to reduce the emissions in vehicles have been made with regulations such as the EURO Emission directives [126]. NO_x_ can be also produced in mammals and it is involved in diverse physiological and pathological pathways. Defects in NO_x_ pathways could be a biomarker for hypertension, cardiac failure, diabetes mellitus, stroke and central nervous system disorder [127].

Different technologies have been developed to detect this family of gases. NO_x_ concentrations have been measured with optical sensors, like infrared laser absorption spectroscopy (with sensitivities in the range of million/trillion by volume) [126] and a photoacoustic sensor (with a detection limit of 450 ppbv) [126]. In addition, SMO based sensors have been studied with materials such as SnO_2_, In_2_O_3_, ITO, ZnO or WO_3_ with sensitivities between tens and hundreds of parts per billion and response times of approx. 10 s [128,129].

A NO_2_ gas sensor that consists of a 750 µm plastic optical fiber and a nanocoating of cadmium sulfide (CdS) was studied in [125]. The coating was fabricated using a chemical bath deposition technique where different exposure times were compared. Concentrations between 0 and 700 ppm were studied in a transmission setup where the amplitude of the spectrum was measured. It shows a good selectivity to NO_2_ (sensitivity of 63.4%) when compared with other gases like CO_2_ (16.2%) or SO_2_ (4.2%). The response time of the sensor was 10 min.

A different NO_2_ sensor was developed using spherical molybdenum disulfide (MoS_2_) and 2D graphene sheets [130] fabricated onto a CRMMF plastic fiber of 650 µm. The sensor was situated in a gas chamber and subjected to NO_2_ concentrations between 0 and 500 ppm and a spectrometer (100–1100 nm) analyzed the changes of the light in a transmission setup. The sensitivity varied depending in the gas concentrations, having the best sensitivity (61%) with 500 ppm. This shows that the sensor was also sensible to acetone ethanol, methane, carbon monoxide and formaldehyde.

Previous works described in this section together with other sensors based on fluorescent [127] or other materials [131] for NO_2_ sensing are summarized in Table 4.

## 7. Conclusions and Outlook

This review gathers valuable information about optical fiber gas sensors based on the LMR phenomenon or with high potential to be used for this purpose. The latter are mainly associated with materials that have been studied separately for LMR generation but not for gas sensing purposes, or are prone to be used for the generation of LMR because the dielectric properties of the material comply with the LMR condition (the real part of the permittivity has to be positive and higher than its own imaginary part and the surroundings).

Collected information has revealed the exceptional properties of the LMR-based optical fiber gas sensors and their versatility using different materials, optical fiber arrangements, fabrication techniques and nanostructures. It is important to note that the LMR technology has not been used in all the gas-sensitive materials referenced in this work. However, the optical properties of all of them are compatible with LMRs, which indicates their great potential and the number of lines of research that are still open.

Currently, a trend can be seen in which the sensitivity increases with the combination of different materials and complex geometric shapes, which is also associated to an increase in the surface area where more gas molecules can interact with the material. Another trend, not only seen in LMR-based optical fiber gas sensors but also in other optical sensors, consists of the utilization of specialty fibers or microstructured fibers, such as D-type, hollow core, suspended core or photonic crystal fibers, among others. Previously mentioned waveguides combined with complex material nanostructures could be exploited in order to obtain sharper resonance wavelength peaks, increase the surface area or improve the mechanical properties of the structure.

Another trend is to use substrates such as coverslips [132] to reduce costs in order to be used at a large scale in fields such as smart cities where the need for sensors to measure parameters related to the quality of air is key in order to act accordingly [133]. This is the objective of the European Commission in their financing of part of this research through the Stardust project.

To conclude, optical fiber gas sensors based on the LMR phenomenon have demonstrated an outstanding performance with ample possibilities of improvement thanks to the recent progress in the fields of photonics and nanotechnology. Nowadays, the development of these sensors is in a research phase where every new research work pushes these devices closer to their final applications. The rapid development and outstanding advantages of LMR technology for gas sensing applications mentioned before reveals a very interesting opportunity and the tremendous potential of these devices in order to fulfil the increasing and highly demanding needs for gas sensors.

## Figures and Tables

**Table 4:** Summary of performance and configuration parameters of RH sensors reviewed in Section 4.

Detected Gas	Material	Deposition Technique	Gas Concentration	Sensitivity	Optical Waveguide	Detection Limit	Reference
H_2_S	(a) ZnO thin-film Zno nanoparticles, (b) Zno nanoparticles (c) SPR (silver +ZnO)	Thermal evaporation technique and nanoparticles dipping	10 to 100 ppm	(a) 1.49, (b) 1.06 and (c) 0.24 nm ppm^−1^	PCS 600 µm	10 ppm *	[30]
H_2_S	(a) ZnO nanorods	Hydrothermal growth method and thermal evaporation coating	10 to 100 ppm	(a) 4.14 nm ppm^−1^ at 10 ppm	PCS 600 µm	10 ppm *	[119]
	(b) ZnO film and nanorods			(b) 2.35 nm ppm^−1^ film at 10 ppm			
H_2_	WO_3_ y Pt	Dip coating	1 vol.% H_2_–99 vol.% N_2_	N/A	200 µm micron quartz-core/plastic-cladding	1 vol.% H_2_–99 vol.% N_2_ *	[21]
H_2_	nanocrystalline SnO_2_	Dip coating	0.1 to 10% H2	N/A	gold-jacketed optical fiber	0.1% *	[123]
H_2_	(a) ITO film and nanoparticles (b) ITO nanoparticles (c) ITO film	Thermal evaporation technique, dip coating for nanoparticles	0 ppm to 100 ppm	(a) 0.71 (b) 0.58 (c) 0.2 nm ppm^−1^	NA 0.37 600 µm	10 ppm *	[121]
H_2_	ZnO nanorods	Growth solution	0.25%	N/A	SMF fiber	0.25% *	[122]
NO	CdTe/CdS quantum dots	Sinalization	10–11 to 10–4 mol/L	0.3412 I/log[(NO)]	Exposed core fiber fabricated in-house	10 pmol/L	[127]
NO_2_	Metallophthalocyanine (MPc)	Thermally deposited	N/A	N/A	Multimode 200 µm	N/A	[131]
NO_2_	CdS	Chemical Bath Deposition (CBD)	0 to 700 ppm	63.4% at 600 ppm	PMMA 750 μm	100 ppm *	[125]
NO_2_	Molybdenum disulfide (MoS_2_)/graphene	Chemical method	0 to 500 ppm	61%	PMMA 650 µm	100 ppm *	[130]

* Data not specified. The values are the minimum concentration found in the articles.
